# Errata

**Published:** 2014-01-31

## Vol. 63, No. 1

In the report, “Recreational Water–Associated Disease Outbreaks — United States, 2009–2010,” an error occurred on page 8 in the ^§^ footnote of the table. The second and third sentences of that footnote should read as follows: “Microcystin was considered a confirmed etiology if water testing detected ≥20 ***μ*g/L** microcystin toxin in water samples collected during or within 1 day of the outbreak exposure period. Microcystin was considered a suspected etiology if water testing detected <20 ***μ*g/L** microcystin toxin in water samples collected during or within 1 day of the outbreak exposure period.”

In the report, “Algal Bloom–Associated Disease Outbreaks Among Users of Freshwater Lakes — United States, 2009–2010,” an error occurred on page 14 in the third sentence of the first full paragraph. That sentence should read as follows: “Microcystin concentrations of ≥20 ***μ*g/L** exceeded the WHO guideline for moderate health risks in four outbreaks (Table 3) (*2*).”

## Vol. 63, No. 2

In the report, “Zinc Deficiency–Associated Dermatitis in Infants During a Nationwide Shortage of Injectable Zinc — Washington, DC, and Houston, Texas, 2012–2013,” on page 35, in the author list, the affiliation footnotes for two authors were incorrect. The author list should read, “Duke Ruktanonchai, MD^1^, Michael Lowe, PhD^1^, Scott A. Norton, MD^2^, Tiana Garrett, PhD^1^, Lamia Soghier, MD^3^, Edward Weiss, MD^4^, June Hatfield, MS^3^, Jeffrey Lapinski, MS^3^, **Steven Abrams, MD^5^**, **Wanda Barfield, MD^6^**” (Author affiliations at end of text). The correct affiliations for the two authors are “**^5^Texas Children’s Hospital, Houston, Texas** and **^6^Div of Reproductive Health, National Center for Chronic Disease Prevention and Health Promotion, CDC**,” respectively.

